# Spatial and Single-Cell Investigations Illuminate Theragnostic value and Immune Landscape of Mitochondrial Dynamin-Like GTPase in Breast Cancer

**DOI:** 10.7150/jca.96100

**Published:** 2024-05-13

**Authors:** Yen-Dun Tony Tzeng, Pei-Yi Chu, Su-Boon Yong, Tzu-Sheng Hsu, Ling-Ming Tseng, Ming-Feng Hou, Jim Jinn-Chyuan Sheu, Jui-Hu Hsiao, Chia-Jung Li

**Affiliations:** 1 Institute of Biomedical Sciences, National Sun Yat-sen University, Kaohsiung 804, Taiwan.; 2 Department of Surgery, Kaohsiung Veterans General Hospital, Kaohsiung 813, Taiwan.; 3 Department of Pathology, Show Chwan Memorial Hospital, Changhua 500, Taiwan.; 4 Department of Allergy and Immunology, China Medical University Children's Hospital, Taichung 404, Taiwan.; 5 Research Center for Allergy, Immunology, and Microbiome, China Medical University Hospital, Taichung 404, Taiwan.; 6 Institute of Molecular & Cellular Biology, National Tsing Hua University, Hsinchu 300, Taiwan.; 7 Comprehensive Breast Health Center, Taipei Veterans General Hospital, Taipei 112, Taiwan.; 8 Division of Breast Surgery, Department of Surgery, Center for Cancer Research, Kaohsiung Medical University Chung-Ho Memorial Hospital, Kaohsiung 807, Taiwan.; 9 Institute of Clinical Medicine, National Cheng Kung University, Tainan 704, Taiwan.; 10 Department of Surgery, Kaohsiung Municipal Minsheng Hospital, Kaohsiung 802, Taiwan.; 11 Department of Obstetrics and Gynecology, Kaohsiung Veterans General Hospital, Kaohsiung 813, Taiwan.; 12 Institute of BioPharmaceutical Sciences, National Sun Yat-sen University, Kaohsiung 804, Taiwan.

**Keywords:** Breast cancer, Spatial transcriptomic, OPA, immune cell, Diagnostic biomarker

## Abstract

**Background:** As we delve into the intricate world of mitochondrial inner membrane proteins, particularly Optic Atrophy types 1 and 3 (OPA1/3), we uncover their pivotal role in maintaining mitochondrial dynamic equilibrium and fusion, crucial for cellular energy production and synthesis. Despite extensive scrutiny, the significance of OPA1/3 in breast cancer (BRCA) and its interplay with the immune microenvironment remain elusive.

**Materials and Methods:** We meticulously sourced BRCA data from renowned repositories such as The Cancer Genome Atlas (TCGA), Genotype-Tissue Expression (GTEx), Gene Expression Omnibus (GEO), and the Human Protein Atlas (HPA), leveraging cutting-edge techniques including single-cell RNA-sequencing (scRNA-seq), spatial transcriptomics, and pharmacogenomics. Through multifaceted data analysis, we endeavored to unravel the intricate role and potential value of OPA1/3 in BRCA tumorigenesis and progression.

**Results:** Our investigation reveals a conspicuous upregulation of OPA1/3 expression in BRCA, correlating with dismal prognoses. Kaplan-Meier plot analysis underscores that heightened OPA1/3 levels are associated with poor survival rates. Both clinical specimens and biobank biopsies corroborate the elevated expression of OPA1/3 in breast cancer patients. Moreover, scRNA-seq unveils a strong correlation between OPA1/3 and macrophage infiltration in the BRCA immune milieu, alongside its association with the cellular communication network involving CXCL, TGFb, VEGF, and IL16.

**Conclusion:** In light of these findings, OPA1/3 emerges as a promising contender for therapeutic targeting and as a potential diagnostic, prognostic, and survival biomarker in BRCA. The implications of our study underscore the pressing need to explore these novel biomarkers to enhance patient outcomes.

## Introduction

Breast cancer remains the most prevalent malignancy among women worldwide, with a rising incidence in younger individuals. Despite advances, drug resistance, recurrence, and metastasis remain major treatment challenges 1. Current treatments include surgery, radiotherapy, and endocrine therapy, with neoadjuvant therapy combining chemotherapy and targeted drugs. Unfortunately, drug resistance often leads to inevitable recurrence, highlighting the urgent need for reliable biomarkers to enhance accurate diagnosis, prognosis, and targeted therapy 1.

The tumor microenvironment encompasses the intricate surroundings within and around tumors, influencing their growth, metastasis, and cellular interactions. Tumor cells exhibit metabolic reprogramming, characterized by abnormal glucose and amino acid uptake, altered nutrient acquisition pathways, and enhanced glycolytic intermediates utilization 2. This metabolic shift leads to the synthesis of essential biomolecules and dysregulated gene expression, impacting the tumor microenvironment 3. Dysregulated glucose, fatty acid, and amino acid metabolism further contribute to tumor progression. Additionally, mitochondrial metabolic products influence immune cell metabolism 4. Elevated levels of reactive oxygen species in tumor cells can damage mitochondrial DNA (mtDNA), disrupting mitochondrial function and ATP production. Consequently, tumor cells predominantly rely on glycolysis for ATP generation, driving metabolic reprogramming 5.

Mitochondria maintain their normal physiological functions by undergoing fusion and fission processes, which allow them to change their position and morphological structure within the cell. Fusion primarily contributes to the synthesis of new mitochondria and repairs damaged ones, such as those with mtDNA mutations or decreased membrane potential. During fusion, damaged mitochondria integrate with healthy ones, facilitating mtDNA repair and membrane potential restoration 6. Under stressful conditions, such as disease or starvation, fusion maximizes ATP production to meet the body's energy demands 7. In mammals, mitochondrial fusion involves three GTPase proteins, including Mfn1 and Mfn2, which regulate fusion of the outer mitochondrial membrane, and OPA1, which controls fusion of the inner mitochondrial membrane 8. Both OPA1 and OPA3 genes encode proteins located in the mitochondrial inner membrane. The OPA1 gene encodes a 960-amino acid dynamin-like mitochondrial GTPase. It consists of 30 exons and produces 8 transcript variants through alternative splicing 9. These variants exhibit tissue-specific expression in humans. Conversely, OPA3 comprises at least three exons that undergo alternative splicing, resulting in two major transcripts: OPA3A and OPA3B 10, 11. While OPA1 is ubiquitously expressed, its main function involves maintaining mitochondrial dynamic balance 12, 13. Specific OPA1 splice variants exhibit independent anti-apoptotic activity by oligomerizing into complexes that regulate apoptotic ridge remodeling 14, 15. Mutations in exon 2 of OPA3 have been associated with optic atrophy, cataracts, ataxia, peripheral neuropathy, and autonomic neuropathy 16. OPA1 mutations impact mitochondrial fusion, energy metabolism, apoptosis control, calcium clearance, and mitochondrial genome integrity maintenance, whereas OPA3 mutations primarily affect energy metabolism and apoptosis regulation.

This study comprehensively analyzed the performance of OPA1/3 in breast cancer, including differential gene expression analysis, protein correlation analysis, pathway analysis, prognostic analysis of different tumor stages, and compared OPA1/3 expression with macrophage infiltration and immunity. Relevance of moderators. The results indicate that OPA1/3 may be an effective prognostic biomarker and are closely related to immune mechanisms, illustrating its potential as an immunotherapeutic agent for a variety of cancers.

## Materials and methods

### Clinical specimens

Paired breast tumor and non-tumor tissues were obtained from 25 breast cancer patient specimens obtained from the Kaohsiung Veterans General Hospital Human Biobank. Written informed consent was obtained from all patients, and the study was approved by the Ethical Governing Committee and conducted in accordance with relevant guidelines and regulations (Approval Code: KSVGH22-003).

### Multi-omics analysis of OPA1/3 in breast cancer

Multi-omics analysis of OPA1/3 in breast cancer encompasses the utilization of diverse bioinformatics tools and platforms. UALCAN, for instance, offers a range of graphical representations including gene expression profiles and survival curves. Additionally, it allows for the evaluation of promoter-centered gene information and enables pan-cancer gene performance analysis 17. Our investigation leveraged UALCAN to delve into patient survival data across various cancer types based on the expression of central genes. Gene Expression Profiling Interactive Analysis 2 (GEPIA2) serves as a widely utilized interactive platform for visualizing gene expression profiles, amalgamating TCGA/GTEx data. In the Tissue Samples section of GEPIA2, a platform offering insights into comparisons between tumor and normal tissues encompassing various genotypes and subtypes, we conducted an analysis of hub gene expression across diverse cancer types. Moreover, we utilized cBioCancer to examine TCGA DNA variants within the OPA1/3 genes, pinpointing their precise genomic locations. Additionally, we explored correlations between gene expression and various clinical variables using data from cBioCancer. Lastly, DriverDBv4, a comprehensive cancer omics database containing a wealth of data ranging from somatic mutations, RNA expression, miRNA expression, protein expression, methylation, copy number variation, to clinical information, along with annotation libraries, was employed in our study 18, 19.

### Spatial transcriptomic sequencing data analysis

Analysis of spatial transcriptome sequencing data began with the quantification of cell counts within individual spots. We then proceeded to examine the expression patterns and spatial distribution of key genes, visually representing our findings. Utilizing various functions, we grouped similar spatial transcriptome data points and performed dimensional reduction. Initially, cluster classification was conducted based on H&E sections, followed by additional cell grouping using reference cell markers. Notably, certain clusters exhibited elevated expression levels of multiple cell markers. To address this, we employed the single-sample gene set enrichment analysis (ssGSEA) algorithm to assess common cell types using the average expression matrix derived from various clusters.

### Analysis of protein levels and immunohistochemistry

The Human Protein Atlas (HPA: https://www.proteinatlas.org) database was utilized to investigate the protein expression levels of OPA1/3 in both human tumors and normal tissues. The study involved analyzing human breast cancer specimens using tissue microarray (TMA) slides obtained from SuperBioChips Laboratories in Seoul, Republic of Korea. These TMA slides included samples collected from individuals with breast cancer, and normal tissues. For immunohistochemistry (IHC), a biotin-free immune enzymatic antigen detection kit (BioTnA, TAHC01D, Kaohsiung, Taiwan) along with specific antibodies was employed to label and detect the target proteins within the tissue samples. Initially, the samples were fixed using 4% paraformaldehyde and permeabilized with Triton X-100 (0.2%). To minimize non-specific antibody binding, 5% BSA was utilized as a blocking agent before the application of specific antibodies. Subsequently, after overnight incubation with primary antibodies at 4°C, secondary antibodies were applied and allowed to incubate at room temperature for 0.5 hours. The TMA slides were examined and evaluated by Li-Tzung Pathology Laboratory (Kaohsiung, Taiwan), and comprehensive slide images were captured using a BX61VS® microscope (Olympus, Tokyo, Japan).

### Assessing the tumor immune cell microenvironment

scRNA-seq has become a powerful tool for investigating cellular diversity and gene expression at the individual cell level. In our study, scRNA-seq data obtained from the GEO repository underwent stringent quality control (QC) using the R package Seurat. This QC process ensured the selection of high-quality cells while minimizing variations across different experimental batches. Using "BiocManager" and the Gene Set Variation Analysis (GSVA) package in R, we identified distinct cell subpopulations through unified manifold approximation and projection (UMAP) clustering. Cell types were annotated by comparing their expression patterns with established cell marker genes using the "SingleR" package in R. Furthermore, we explored the relationship between OPA1/3 expression and genetic markers associated with tumor-infiltrating immune cells. To achieve this, we utilized the TIMER2 database and CIBERSORT method to assess the correlation between OPA1/3 expression and immune infiltration. Correlations were calculated using the Pearson correlation coefficient for various cell types, including CD4+ T cells, CD8+ T cells, neutrophils, macrophages, eosinophils, and natural killer cells. Additionally, ESTIMATE was employed to evaluate the correlation between OPA1/3 expression and ESTIMATE score, immune score, and stromal score 20

### Statistical analysis

Statistical analysis was performed using R software (version 4.0.2). Descriptive statistics are expressed as mean ± standard deviation (SD). Differences were considered statistically significant at p < 0.05. Additionally, various statistical tests were employed to assess the significance of the findings, including [mention specific tests or methods if applicable].

## Results

### Genetic Landscape and Functional Analysis of OPA1 and OPA3 in Breast Cancer

**Figure 1:**
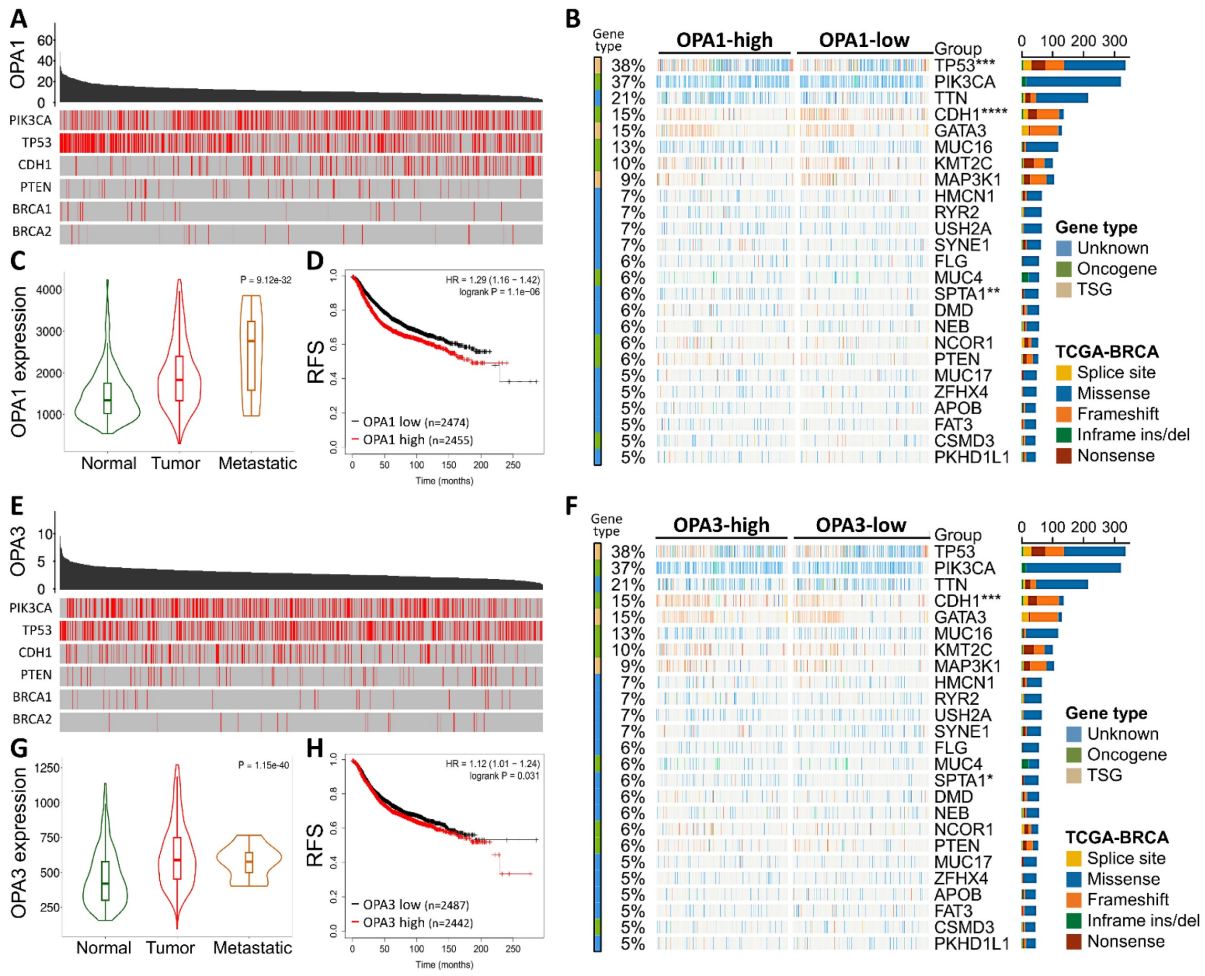
The comprehensive analysis of the frequency and distribution of mutations in the OPA1 and OPA3 genes across breast cancer. (A) Examination of mutations across a diverse range of cancer types reveals the full spectrum of genetic alterations affecting the OPA1 and OPA3 genes. (B & C) Investigation into the relationships among highly mutated genes in OPA1 and OPA3 underscores potential interactions and co-occurrences, with specific mutation sites highlighted to enable thorough examination. (D & E) Utilizing Fisher's exact test to compare mutation frequencies across different expression levels of OPA1 and OPA3 provides valuable insights into the precise distribution of mutations within each expression group.

Initially, our investigation delved into the mutational landscape of OPA1 and OPA3 genes within breast cancer (BRCA), meticulously scrutinizing their interplay with other established cancer-related genes such as PIK3CA, TP53, CDH1, PTEN, BRCA1/2, among others (Fig. 1B&E). Subsequently, leveraging data from The Cancer Genome Atlas (TCGA) database, we extracted information on the top 25 genes affected by both high and low expression levels of OPA1/3 in BRCA patients, which we visualized using a waterfall chart (Fig. 1B&F). Furthermore, we conducted a comprehensive assessment of OPA1/3 expression across different clinical stages, revealing a notable increase in their expression levels in both tumor tissues and advanced-stage patients (Fig. 1C and G). To discern the prognostic implications of OPA1/3 expression, we employed Kaplan-Meier plots to analyze its impact on Relapse-free survival (RFS) in BRCA patients. Our findings underscored a significant association between high OPA1/3 expression and unfavorable prognosis (Fig. 1D and H).

### Overexpression of OPA1/3 Promotes Breast Cancer Progression

**Figure 2:**
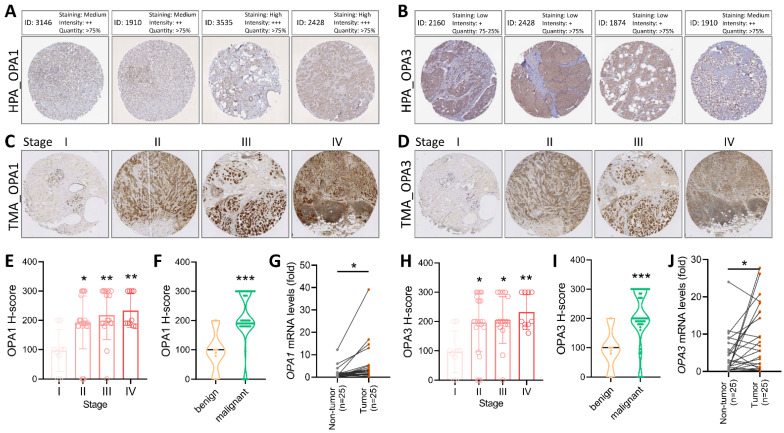
Delves into the pathological alterations of OPA1 and OPA3 in human breast cancer. (A-B) Immunohistochemical evaluation of OPA1 and OPA3 protein expression levels in breast cancer samples sourced from diverse patients using data retrieved from the Human Protein Atlas database. (C-D) Representative images depicting the expression of OPA1 and OPA3 at varying staining intensities in breast cancer tissues. (E-H) Comparative analysis of OPA1 and OPA3 expression at different stages of breast cancer progression, presented through box plots. (F-I) Violin plots illustrating the expression profiles of OPA1 and OPA3 in both benign and malignant breast cancer tissues. (G-J) Quantitative PCR (qPCR) analysis of OPA1 and OPA3 expression in 25 paired breast cancer (T) and non-tumor (N) tissue samples to determine their expression levels in tumor and adjacent non-tumor tissues. * P < 0.05, ** P < 0.01, *** P < 0.001.

To validate the comprehensive analysis conducted, we proceeded to investigate the protein levels of OPA1/3 in breast cancer utilizing data from the Human Protein Atlas (HPA) database. The analysis revealed elevated levels of OPA1/3 in breast cancer specimens (Fig. 2A&B). Subsequently, we conducted immunohistochemistry (IHC) analysis on tumor tissues using a commercial breast cancer tissue microarray (TMA) to assess OPA1/3 expression (Fig. 2C&D). The IHC results demonstrated a significant increase in OPA1/3 levels as breast cancer advanced and became more malignant with higher stages (Fig. 2E&H), corroborating the observations from the HPA database. Furthermore, the H-score analysis revealed abundant OPA1/3 expression across various cancer stages and in both benign and malignant tumors, indicating a notable upsurge with tumor progression (Fig. 2F&I). Subsequently, we examined the mRNA expression of OPA1/3 in 25 pairs of BRCA and non-tumor tissues sourced from the KSVGH Biobank. The quantitative PCR (qPCR) results exhibited a significant upregulation of OPA1/3 in BRCA tissues compared to non-tumor tissues (Fig. 2G&J).

### Identification of Heterogeneous BRCA Cell Subpopulations Expressing OPA1/3

**Figure 3:**
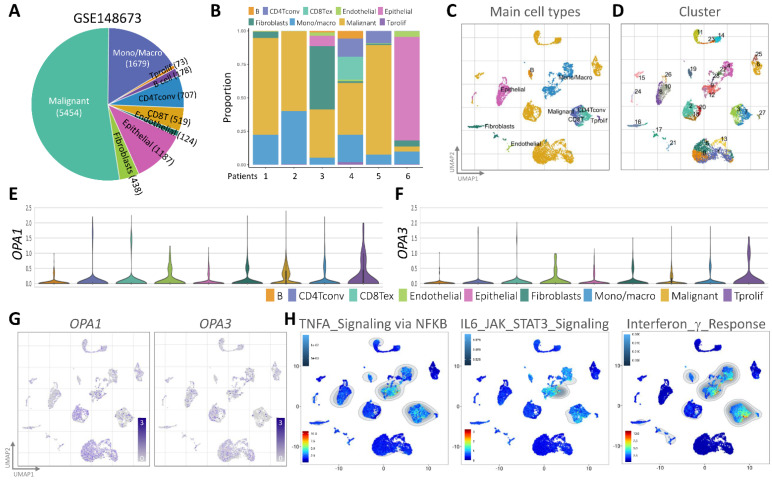
scRNA-seq analysis for external validation. (A-B) Depiction of immune cell population proportions from scRNA-seq analysis data. (C-D) Cell clusters categorized by color according to main cell types and distinct clusters. (E-F) Transcript levels of OPA1 and OPA3 in various cell clusters illustrated using violin plots. (G) Presentation of 28 cell subpopulations obtained after downscaling and cell aggregation, showcasing the expression and distribution of candidate genes. (H) Functional gene set enrichment analysis (GSEA) outcomes, including TNFA signaling via NFKB, IL6/JAK/STAT3 signaling, and interferon gamma response.

In our quest to unravel the intricate landscape of intratumoral heterogeneity within BRCA and to gain insights into the dynamics of the tumor microenvironment, we initiated our exploration by analyzing scRNA-seq data. This analysis was conducted using the GSE148673 dataset, wherein we employed the Seurat R suite to process the samples, identifying the top 9 principal components across all clusters for cell subpopulation delineation (Fig. 3A). To visually represent the distribution of cells from six patients across different clusters, we depicted this information in a histogram (Fig. 3B). Subsequently, leveraging the Unified Manifold Approximation and Projection (UMAP) technique, we classified cells into 9 distinct clusters, examining their characteristics and differences based on major cell types and subclusters (Fig. 3C&D). Further, we meticulously assessed the RNA levels of OPA1/3 in each cell within the diverse clusters (Fig. 3E&F). By identifying genes expressing OPA1/3 within each cluster, we pinpointed cell type-specific markers crucial for subsequent cell type classification (Fig. 3G). Following a thorough analysis of OPA1/3 expression and distribution in these scRNA-seq datasets, we proceeded to perform gene set enrichment analysis (GSEA) to probe the potential interplay between OPA1/3 and key signaling pathways, including TNFA signaling, IL6/STAT3 signaling, and interferon gamma response (Fig. 3H).

### Examining the tumor microenvironment and the pathological relevance of OPA1/3 through spatial transcriptomics analysis

**Figure 4:**
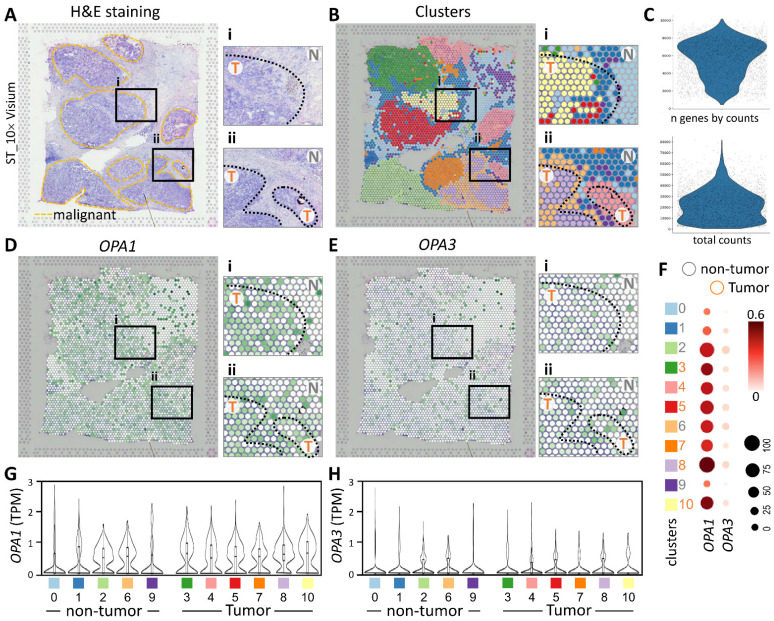
Spatial transcriptomes define the gene expression and distribution of different cell clusters. (A-B) Spatial transcriptomics allowed precise alignment of clusters with morphological features, demarcating malignant regions with yellow dashed outlines and highlighting genetic variants. (C) Dataset characteristics include total and gene counts. (D) Using 10x Visium spatial gene expression, OPA1 and OPA3 genetic changes across tissue sections were examined. (F) Dot plots display gene expression levels in clusters. (G-H) Violin plots summarize OPA1 and OPA3 transcript levels in tumors and non-tumors.

In this study, we utilized spatial transcriptomics techniques to investigate the gene-immune landscape of OPA1/3 in human breast cancer tumors (PMID: STDS0000027). By integrating transcriptome signatures onto H&E-stained histological sections, we annotated clusters based on hematoxylin and eosin staining and cellular markers, enabling the identification of distinct tumor morphological regions (Fig. 4A). A detailed analysis revealed 3,798 spots and 36,601 genes in the sample, each displaying a unique expression signature. The spatial distribution of these signatures was depicted on the tumor histological image using local barcoding (Fig. 4B). Utilizing gene counts and total counts, we generated violin plots to visualize gene expression distribution, assess sample quality, and normalize the data effectively (Fig. 4C). Subsequent examination of OPA1/3 transcript levels revealed abundant OPA1 expression within the tumor area, with OPA3 expression, albeit lower than OPA1, also concentrated in tumor regions (Fig. 4D&E). Furthermore, Figure 4F-H illustrates variations in OPA1 and OPA3 transcript levels between tumor and non-tumor areas, as well as their distribution across 10 different cell clusters. This comprehensive spatial transcriptomics analysis provides valuable insights into the tumor microenvironment and highlights the potential pathological significance of OPA1/3 in breast cancer.

### Deciphering breast tumor microenvironment through intercellular communication network analysis

**Figure 5:**
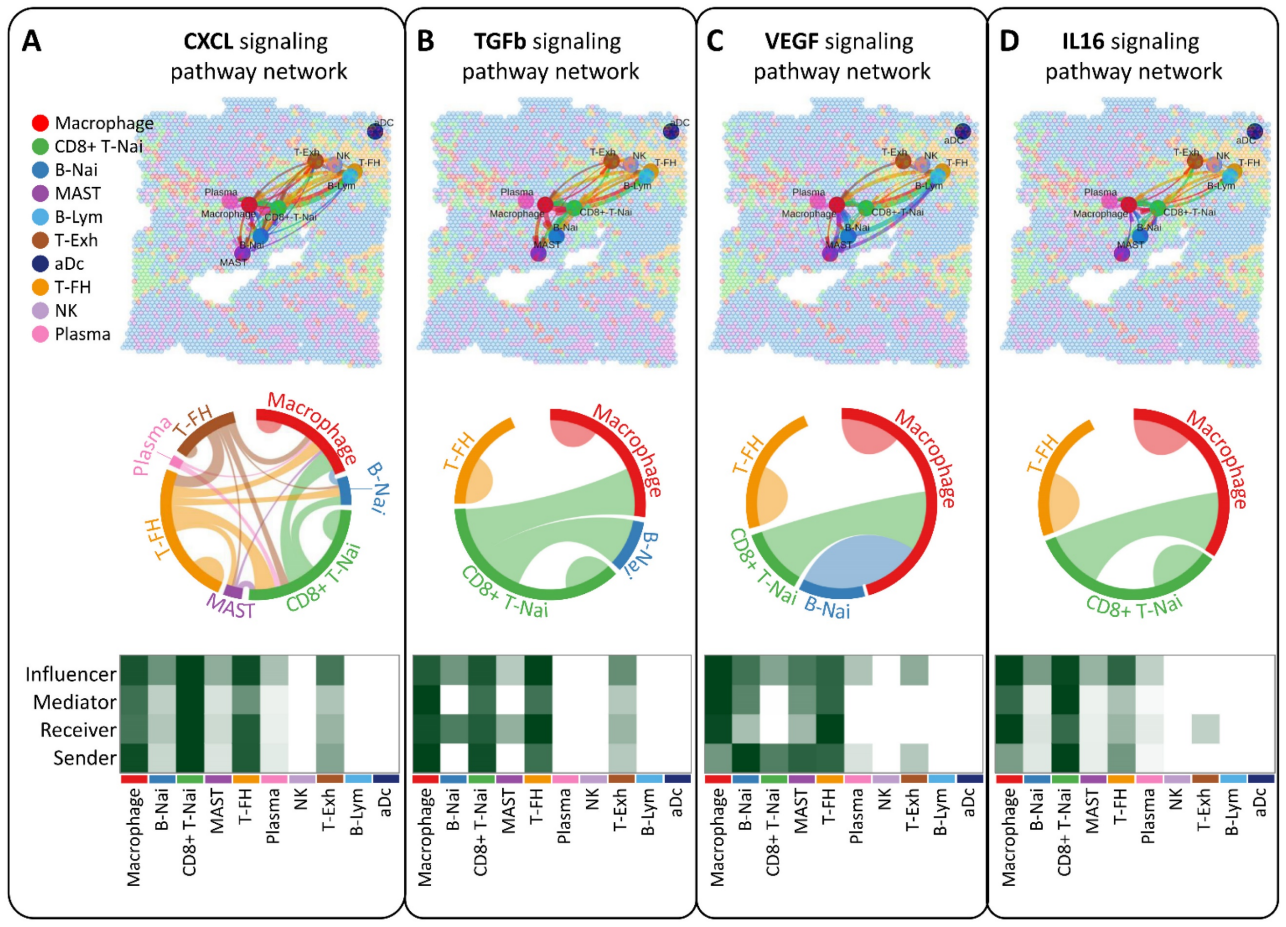
Analysis of intercellular communication networks among various cell types using CellChat. Spatial transcriptomics is employed to deduce distinct intercellular communications within various signaling pathway networks. Network centrality scores for the CXCL (A), TGFb (B), VEGF (C), IL6 (D) signaling pathway are determined for each cell type. Heatmaps illustrate the signaling effects of cell populations and display network centrality scores for different signals.

Our study aimed to elucidate the interplay of ligands and receptors between cells within the breast tumor microenvironment using CellChat, focusing on CXCL, TGFb, VEGF, and IL16 signaling pathways pivotal in intercellular communication (Figure 5A-D). Across ten cell types, including CD8+ T-Nai, B-Nai, T-FH, MAST, macrophages, B-Lym, T-Exh, NK, Plasma, and aDc, macrophage clusters emerged as key regulators, exhibiting robust signaling interactions among senders, receivers, mediators, and influencers (Figure 5A-D, bottom panel). This ligand-receptor-mediated communication underscores the potential involvement of diverse cell types in breast cancer development.

**Figure 6:**
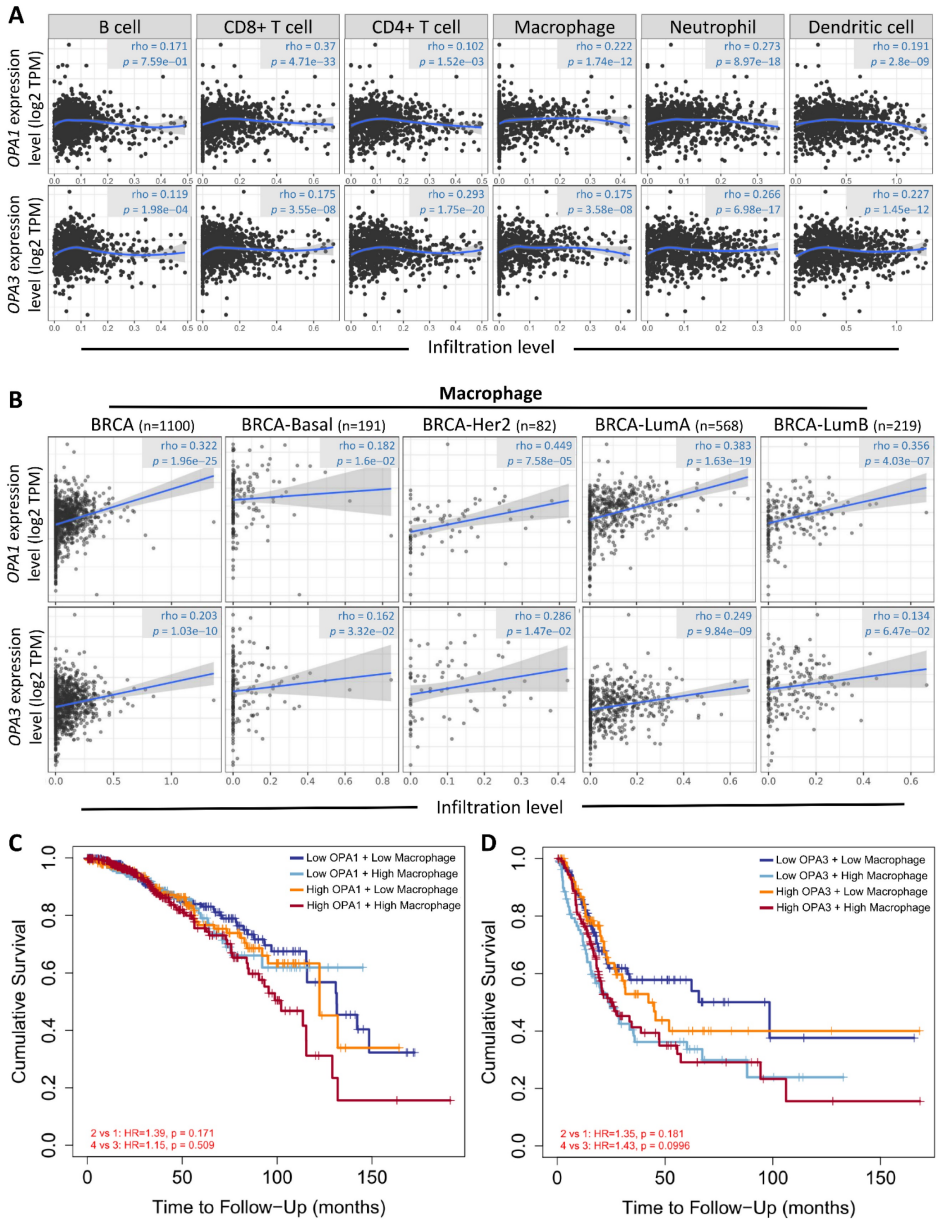
Examining the relationship between prognostic gene expression and immune cell infiltration. (A) Investigating the correlation between OPA1 and OPA3 expression levels and the infiltration of various immune cells within tumors. (B) Evaluating the association between OPA1 and OPA3 expression and the extent of macrophage infiltration. (C) Illustrating the survival discrepancies among macrophages based on different levels of OPA1 and OPA3 expression using a Kaplan-Meier plot.

Drawing from these results, we postulated a strong correlation between the examined sample and macrophages, considering the established connection between breast cancer advancement and the immune microenvironment. To explore this relationship further and understand its regulatory mechanisms, we conducted analyses using the Gene Immunity Database. Our analysis revealed OPA1/3's association with various immune cells, particularly CD4+ T cells, CD8+ T cells, B cells, neutrophils, macrophages, and myeloid dendritic cells (Fig. 6A). Notably, macrophages exhibited significant enrichment and infiltration within tumor tissues, correlating with elevated OPA1/3 expression levels. Moreover, there was a positive correlation between macrophage infiltration and OPA1/3 expression in different BRCA subtypes characterized by heightened OPA1/3 levels (Fig. 6B). Patients with elevated OPA1 or OPA3 expression coupled with high macrophage infiltration exhibited shorter survival times compared to those with high gene expression alone (Fig. 6C-D). These findings strongly suggest that OPA1/3 may promote BRCA development and progression by modulating immune cell infiltration.

### Identifying potential drugs targeting OPA1/3 through pharmacogenomic analysis

**Figure 7:**
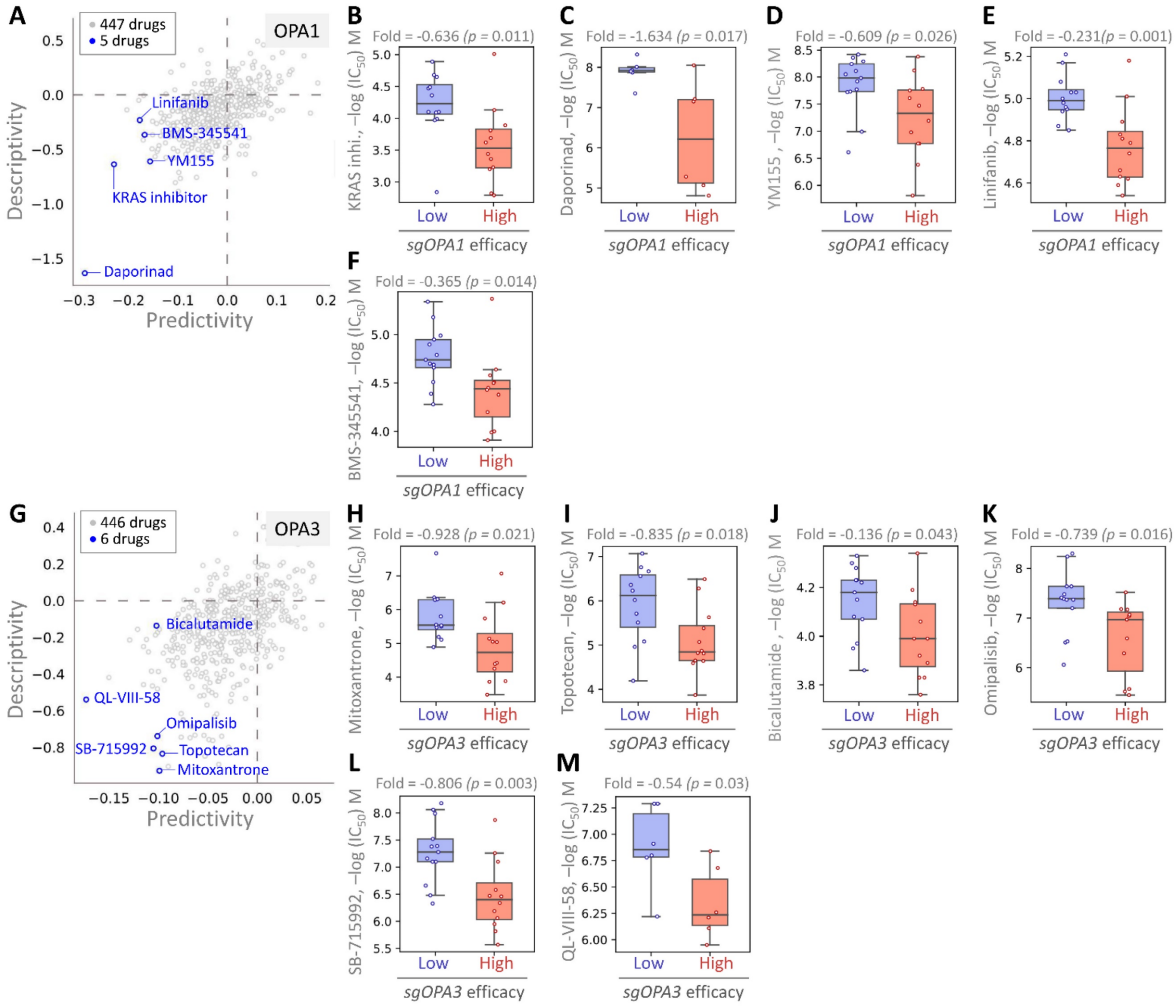
Assessing drug responsiveness and pharmacogenomics in breast cancer cells. (A & G) Genetic signatures are screened using pharmacogenetic databases to identify potential drug candidates. Drug sensitivity of sgOPA1 (B-F) and sgOPA3 (H-M) gene knockout BRCA cell lines to small molecules is evaluated, highlighting maximum inhibitory concentrations.

Finally, we employed pharmacogenomic libraries to pinpoint small molecule drugs that exhibit heightened efficacy in the presence of elevated OPA1/3 expression, thereby uncovering potential therapeutic options against BRCA with high OPA1/3 levels. Through cross-correlation analysis between drug response and CRISPR knockdown of OPA1/3 in various BRCA cell lines using single guide RNA (sgRNA), we scrutinized the effects of 447 drugs on sgRNA-mediated OPA1 inhibition in BRCA cells (Fig. 7A). This analysis unveiled five small molecule drugs, with the first four demonstrating potent inhibition: KRAS, Daporinad, YM155, Linifanib, and BMS-345541, exhibiting altered potency (Fig. 7B-F). Furthermore, for OPA3, we screened 446 drugs and identified six with inhibitory effects, with six small molecule drugs demonstrating significant inhibitory effects: Mitoxantrone, Topotecan, Bicalutamide, Omipalisib, SB-715992, and QL-VIII-58 (Fig. 7G-L). These findings suggest the potential utility of these drugs as anticancer agents targeting OPA1/3 to modulate breast cancer cell development.

Moreover, we extended our analysis to explore the molecular mechanisms underlying the interactions between these identified drugs and OPA1/3 expression in BRCA cells. By investigating the downstream signaling pathways affected by these drugs and their impact on cellular processes such as proliferation, apoptosis, and metastasis, we aim to gain deeper insights into their therapeutic potential and mechanisms of action. Additionally, further validation studies, including *in vitro* and *in vivo* experiments, are warranted to confirm the efficacy and safety of these drugs as potential treatments for BRCA with elevated OPA1/3 expression. Overall, our pharmacogenomic analysis provides a foundation for the development of personalized therapeutic strategies targeting OPA1/3 in breast cancer patients.

## Discussion

The intricate interplay of mitochondrial morphological dynamics, encompassing fusion and fission mechanisms, significantly influences mitochondrial function 21. This equilibrium between fusion and fission processes is pivotal in cancer pathogenesis 22. Positioned within the inner mitochondrial membrane (IMM), OPA1 plays a crucial role in preserving mitochondrial fusion and cellular well-being 23. By modulating the delicate equilibrium between mitochondrial fission and fusion, OPA1 regulates mitochondrial dynamics.

Studies have indicated that OPA1 is overexpressed or mutated across various cancers and is closely associated with protein phosphorylation, patient prognosis, and immune cell infiltration 24. Additionally, research has shown concurrent amplification of OPA1 and MFN1 copy numbers, synergistically activated in tumor epithelial cells in lung cancer tissues. Both OPA1 and MFN1 exhibit high expression levels in LUAD tumor tissues, with elevated OPA1 expression correlating with poor prognosis. Intriguingly, OPA1 deficiency disrupts mitochondrial dynamics, impeding respiratory function, and rendering tumor epithelial cells more sensitive to CD8+ T cell activity in non-small cell lung cancer 25. Mechanistically, OPA1 knockdown alters cristae morphology and inhibits electron transport chain assembly and activity, resulting in impaired tumor cell proliferation due to decreased NAD+ regeneration. Concurrent inhibition of Drp1 and Opa1 restores cristae morphology, electron transport chain activity, and cell proliferation, highlighting the role of mitochondrial fission in driving electron transport chain dysfunction induced by Opa1 knockdown 26. Moreover, CHD6 knockdown in colorectal cancer cells decreases mitochondrial length and cristae number, ultimately triggering apoptosis 27. Mitochondrial fusion not only enhances cancer cell proliferation but also aids in mitochondrial repair and preservation of mitochondrial DNA integrity by diluting damaged proteins under oxidative stress conditions 28.

Limited research has been conducted on the role of OPA3 in tumor progression and the tumor microenvironment. A study examining ovarian cancer revealed elevated OPA3 expression in ovarian cancer tissues and cells compared to normal ovarian tissues/cells, with high OPA3 levels correlating with poor overall survival. Additionally, OPA3 exhibited a strong association with immune infiltration in ovarian cancer, displaying significant correlations with various immune marker panels 25. Similarly, heightened OPA3 expression was observed in pancreatic cancer tissues. Knockout of the OPA3 gene led to a notable decrease in energy metabolism, evidenced by reduced oxygen consumption rate and cellular ATP content, resulting in inhibited cell proliferation and decreased expression of epithelial-mesenchymal transition (EMT) markers 29. In hepatocellular carcinoma (HCC), OPA3 was markedly upregulated and associated with unfavorable prognosis. Knockdown of OPA3 hindered the growth of HCC cells both *in vitro* and *in vivo*, accompanied by reduced glucose uptake, lactate production, intracellular ATP levels, and extracellular acidification rate 30.

The incidence of breast cancer continues to rise, with an alarming trend of affecting individuals at younger ages. Detecting and treating breast cancer early are crucial for improving patient outcomes, highlighting the urgent need for sensitive biomarkers. However, our literature search found that there are currently no reports of OPA1/3 in breast cancer, so it is necessary to conduct a comprehensive analysis of OPA1/3 in breast tumors. This study conducted a comprehensive analysis using multi-omics data to investigate the expression profiles of OPA1/3 in breast cancer patients and their potential diagnostic and prognostic value. The findings revealed a significant increase in both transcriptional and protein levels of OPA1/3 within breast cancer tissues, showing strong correlations with various clinical parameters. Further validation from biopsies obtained from a human biobank confirmed the heightened expression of OPA1/3 specifically in tumor tissues. Kaplan-Meier analysis provided additional insights, demonstrating a notable association between OPA1/3 mRNA expression levels and patient survival time. Moreover, multivariate analysis uncovered intriguing connections between OPA1/3 mRNA expression levels and macrophage infiltration. This association may be mediated through the CXCL, TGFb, VEGF, and IL16 signaling pathways, shedding light on the intricate interplay between mitochondrial dynamics and the tumor microenvironment in breast cancer progression.

## Conclusion

To summarize, our systematic multi-omics analysis of OPA1/3's performance and prognostic implications in BRCA using bioinformatics tools sheds light on its immunological and biological roles in this context. These insights suggest that OPA1/3 could serve as valuable targets and biomarkers for personalized BRCA treatment strategies, thereby offering the potential to enhance diagnostic and therapeutic approaches for BRCA patients and improve overall prognosis. However, further investigation is needed to fully elucidate their mechanisms of action in tumor formation and progression and to develop targeted therapeutic interventions.
